# Effects of Vibration Rolling with and without Dynamic Muscle Contraction on Ankle Range of Motion, Proprioception, Muscle Strength and Agility in Young Adults: A Crossover Study

**DOI:** 10.3390/ijerph17010354

**Published:** 2020-01-04

**Authors:** Bo-Jhang Lyu, Chia-Lun Lee, Wen-Dien Chang, Nai-Jen Chang

**Affiliations:** 1 Department of Sports Medicine, Kaohsiung Medical University, Kaohsiung 807, Taiwan; jison0309@gmail.com; 2 Center for Physical and Health Education, National Sun Yat-sen University, Kaohsiung 804, Taiwan; karenlee1129@gmail.com; 3 Department of Sport Performance, National Taiwan University of Sport, Taichung 404, Taiwan; changwendien@ntupes.edu.tw; 4 PhD Program in Biomedical Engineering, Kaohsiung Medical University, Kaohsiung 807, Taiwan; 5 Regenerative Medicine and Cell Therapy Research Center, Kaohsiung Medical University, Kaohsiung 807, Taiwan

**Keywords:** sports, performance, vibration therapy, self-myofascial release, therapeutic exercise

## Abstract

Vibration rolling (VR) has emerged as a self-myofascial release (SMR) tool to aid exercise performance when warming up. However, the benefits of VR on exercise performance when combined with dynamic muscle contraction are unclear. The purpose of this study was to investigate the immediate effects of the combination of VR with dynamic muscle contraction (DVR), VR, and static stretching (SS) during warm-up on range of motion (ROM), proprioception, muscle strength of the ankle, and agility in young adults. In this crossover design study, 20 recreationally active adults without musculoskeletal disorders completed three test sessions in a randomized order, with 48 h of rest between each session. Participants completed one warm-up intervention and its measurements on the same day; different warm-up interventions and measurements were performed on each of the three days. The measurements included ankle dorsiflexion and plantarflexion ROM, ankle joint proprioception, muscle strength, and agility. After DVR and VR intervention, ankle dorsiflexion ROM (both DVR and VR, *p* < 0.001), plantarflexion ROM (both DVR and VR, *p* < 0.001), plantar flexor muscle strength (DVR, *p* = 0.007; VR, *p* < 0.001), and agility (DVR, *p* = 0.016; VR, *p* = 0.007) significantly improved; after SS intervention, ankle dorsiflexion and plantar flexion ROM (dorsiflexion, *p* < 0.001; plantar flexion, *p* = 0.009) significantly improved, but muscle strength and agility were not enhanced. Compared with SS, DVR and VR significantly improved ankle plantar flexor muscle strength (*p* = 0.008 and *p* = 0.001, respectively). Furthermore, DVR significantly improved ankle dorsiflexion compared with VR (*p* < 0.001) and SS (*p* < 0.001). In conclusion, either DVR, VR, or SS increased ankle ROM, but only DVR and VR increased muscle strength and agility. In addition, DVR produced considerable increases in ankle dorsiflexion. These findings may have implications for warm-up prescription and implementation in both rehabilitative and athletic practice settings.

## 1. Introduction

The ankle joint is the most common site of injury in active individuals [1]. The risk of acute lower extremity injuries can be reduced through warm-up regimens [2]. In addition, an individual’s exercise performance can be improved through various warm-up programs such as range of motion (ROM) or muscle strength [3,4]. Several warm-up techniques can be performed, including static stretching (SS), dynamic stretching, proprioceptive neuromuscular facilitation, foam rolling (FR), and vibration therapy. However, the optimal warm-up method has not been ascertained yet. SS prior to exercise is a common practice to reduce muscle stiffness and increase ROM, but, recently, numerous studies have reported that SS may negatively affect sports performance as a result of changed joint instability or altered optimal intramuscular viscoelastic properties [5,6]. Alternatively, FR has become one of the most popular self-myofascial release (SMR) tools [7]. FR for SMR could improve ROM while increasing blood flow and circulation to the soft tissues [5,8]. Individuals use their own body weight to apply pressure to target tissues. Recently, a systematic review reported that, as an active warm-up exercise, FR may reduce muscle stiffness, increase ROM, and enhance pre- and postexercise muscle performance [9]. A meta-analysis summarized evidence supporting the validity of the widespread use of FR as a warm-up activity [10]. In addition, FR reduces delayed-onset muscle soreness (DOMS) and increases the pressure pain threshold (PPT); thus, it may optimize recovery from training [7,11,12]. However, some studies have indicated that FR seems to have no beneficial effect on maximum voluntary contraction force [8] and muscle strength [13]. Notably, FR with active joint motion has a greater effect on passive joint ROM and PPT than rolling without motion; FR with active joint motion may modulate the activity of the antagonistic muscle through reciprocal inhibition [14]. This suggests that FR with dynamic muscle contraction has greater benefits than traditional SMR techniques.

Vibration therapy is an alternative warm-up method; it can increase flexibility [15], improve balance [16], and reduce postexercise DOMS [17,18], effects which are attributed to the enhancement of reflex activity by the stimulation of the muscle spindle Ia to initiate a tonic vibratory reflex [19,20] and increased intramuscular temperature [21]. Recently, vibration and rollers have been combined to develop vibrating foam rollers. However, little is known about the effectiveness of vibration rolling (VR), particularly for the ankle joint. Lim et al. showed that ROM after VR (1 min × 5 sets on both hamstrings) was significantly increased compared with that after FR [22]. Romero-Moraleda et al. demonstrated that after exercise with induced muscle damage, VR (1 min × 5 sets on both quadriceps) more significantly improved PPT and ROM than FR [23]. Cheatham demonstrated that compared with a nonvibrating roller, a vibrating foam roller (33 Hz, 2 min on quadriceps) significantly increased PPT and knee joint motion [24]. Lee et al. demonstrated that VR (28 Hz, 30 s × 3 sets on hamstrings and quadriceps) significantly increased the ROM of knee flexion and extension, and isokinetic peak torque, muscle strength, and dynamic balance were also increased [25]. Therefore, most studies investigating the immediate effects of VR have mainly focused on the knee joint, and most outcome measurements have been determined by ROM and PPT; however, little is known about exercise performance (e.g., agility).

The ankle joint is the most common site of injury and a critical factor of the kinetic chain in active individuals. To date, we found only two peer-reviewed studies that investigated the immediate effect of warm-up VR (49 Hz, 20 s × 3 sets on calf muscles) on the ankle joint [26] and modeled recovery after an induced fatigue protocol [27]; however, the primary outcome tested was ROM. Therefore, the benefits of VR and dynamic muscle contraction on ankle ROM, proprioception, muscle strength, and agility exercise performance in young adults are unclear, and a gap exists in our knowledge of the efficacy of this methodology. Accordingly, in this study, we hypothesized that VR can generate an SMR effect on targeted calf muscles and thus increase ROM. In addition, VR can generate positive effects on ankle joint proprioception, maximal muscle strength, and agility through the facilitation of vibration-induced neuromuscular activation [25,28]. Moreover, dynamic muscle contraction may increase blood flow to enhance muscle contraction [29] and modulate activity of the antagonistic muscle through reciprocal inhibition to increase flexibility [14], thereby aiding exercise performance.

Therefore, the purpose of this study was to investigate the immediate effects of the combination of VR with dynamic muscle contraction (DVR), VR, and SS during warm-up on ROM, proprioception, muscle strength of the ankle, and agility in young adults. The primary outcome was ankle ROM. The secondary outcomes were assessed using ankle joint proprioception, muscle strength, and agility tests.

## 2. Materials and Methods

### 2.1. Participants

The study protocol was approved by the Kaohsiung Medical University Hospital Institutional Review Board (KMUHIRB-F(I)-20190061). Twenty male students (age: 21 ± 1.01 year, body mass: 68.45 ± 9.82 kg, height: 172.9 ± 6.04 cm; body mass index: 22.8 ± 2.54). The inclusion criteria were recreationally active adults aged 20–40 years without any musculoskeletal disorders in the 6 months before the study. “Recreationally active” was defined as performing exercise approximately 2–3 times per week [30]. The exclusion criteria were cardiovascular or respiratory diseases, contraindications to exercise (e.g., neuromusculoskeletal injury or lower back pain), muscle strain and ligament sprain, joint instability or laxity in the lower extremity, head or spinal injury, and visual, vestibular, or balance disorders 6 months before the study. All participants were informed regarding the benefits and risks of this study, and written informed consent was obtained from all participants.

### 2.2. Study Procedures

**Figure 1:**
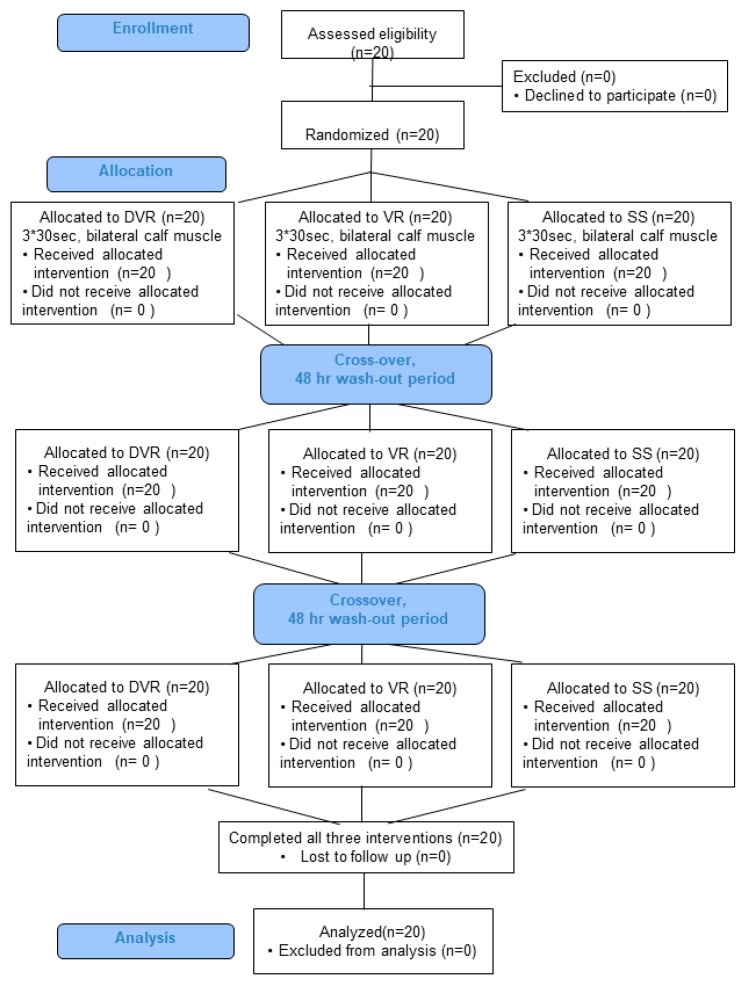
CONSORT flow diagram of the experimental design.

This study was a crossover study. Tests performed by each participant were assessed in a laboratory at the Department of Sports Medicine, Kaohsiung Medical University. Prior to the assessment session, participants underwent a familiarization session in which they were instructed on how to perform DVR, VR, and SS exercises. During this orientation, participants were familiarized with the procedures and practiced with the assessment tools and equipment of the study. One day after the familiarization session, each participant completed three test sessions in a randomized order, with 48 h of rest between each session (Figure 1). A researcher prepared randomly shuffled cards (A card: DVR; B card: VR; and C card: SS) and sealed each card in an opaque envelope. Each participant drew an envelope and opened the envelope to discover the exercise assignment. Participants were requested to avoid strenuous activities 24 h before each test session. Upon arriving at the laboratory, they were asked to perform heel up and down exercises 10 times in two sets before the pretest in each test session [26,31]. Subsequently, participants were assessed by flexibility tests (i.e., ROM of ankle dorsiflexion and plantarflexion), isokinetic strength and ankle joint proprioception tests, and a figure-8 hop test. After completion of pretest assessments, each participant drew a randomly shuffled card that specified one of three interventions (DVR, VR, or SS), then performed that intervention for a 4-min session. The protocols for these exercises are provided later in detail. Immediately after the intervention, posttest assessments were conducted in the same order as pretest measures. Participants completed one warm-up intervention and its measurements on the same day; different warm-up interventions and measurements were performed on each of the three days. The flowchart of the experimental design is shown in Figure 1.

### 2.3. Outcome Measures

#### 2.3.1. Primary Outcomes

The angle of ankle dorsiflexion and plantarflexion in the supine position was measured using a plastic goniometer, which is commonly used by clinical practitioners [32]. Participants were asked to actively dorsiflex and plantar flex their ankle as much as possible. The average measurement of two trials was recorded. In this study, intraclass correlation coefficients (ICCs) were 0.921 (dorsiflexion) and 0.955 (plantar flexion), indicating excellent test–retest reliability. In addition, the minimum detectable change (MDC) value was calculated as 1.27° (dorsiflexion) and 1.00° (plantar flexion), corresponding to the standard error of measurement (SEM) of 0.46 (dorsiflexion) and 0.36 (plantar flexion).

#### 2.3.2. Secondary Outcomes

**Figure 2:**
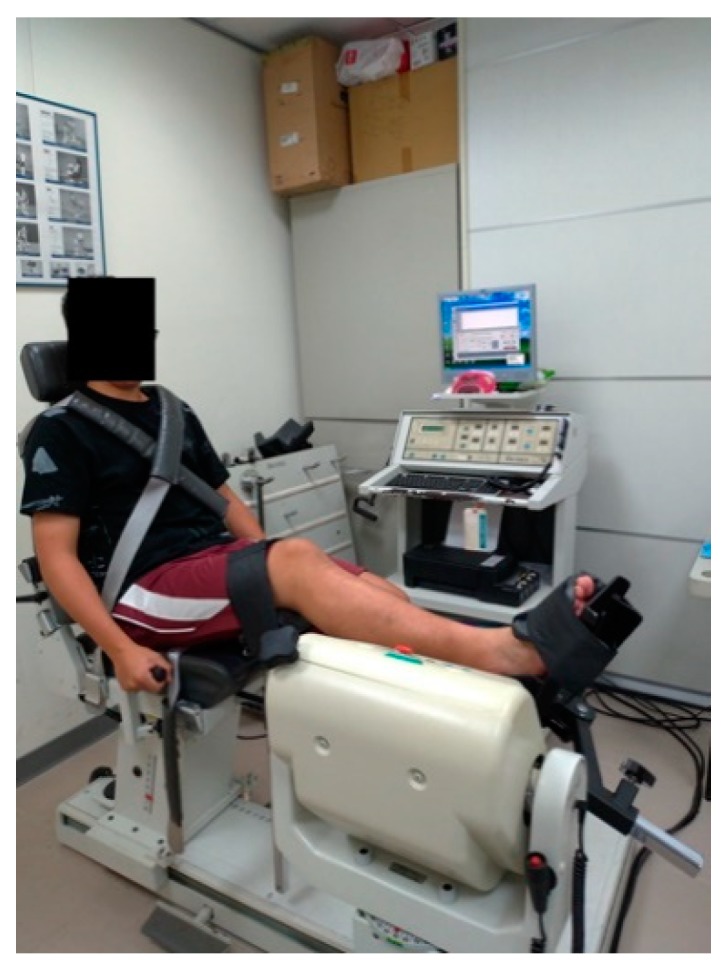
Biodex isokinetic dynamometer for ankle testing.

Ankle joint proprioception, muscle strength, and agility were assessed. Regarding ankle joint proprioception tests, the Biodex isokinetic dynamometer (Biodex System 3 Pro, New York, NY, USA) was used to measure ankle joint proprioception and ankle muscle strength [33]. For the ankle joint proprioception test, participants sat in an upright position on the Biodex dynamometer chair with their torso, right thigh, and right ankle stabilized by straps to minimize compensatory body movements. The right ankle was positioned on the dynamometer. Each participant wore an eye mask to eliminate the visual feedback input (Figure 2). Next, participants actively moved their ankle to the target angle of 30° of plantar flexion for 5 s to memorize the position and then returned to 0° of the ankle neutral position. Participants were asked to move their ankle to that angle again through active contraction without being able to see their ankle; participants reached the target angle and then stopped. The average of three tests was recorded. The difference (absolute error) between the setting and reproduced angle (perceived angle) was recorded as the index of proprioception. Isokinetic ankle muscle strength measurements were conducted with participants in the same position, but the eye mask was removed. Participants were asked to actively perform ankle dorsiflexion and plantar flexion as fast as they could two times at a set angular velocity of 60°/s. The peak torque in N·m was recorded using Biodex software, and the highest torque value was used in statistical analysis. The peak torque was normalized to body weight.

Regarding agility assessment, we used the figure-of-8 hop test as an agility test [34]. Reliability for this test was excellent, with an ICC of 0.95 (SEM, 1.66 s) [35]. In this test, participants hopped twice as quickly as possible around a 5-m course on the test foot in a figure 8. The time taken for a participant to complete two circuits of the figure 8 was measured using a stopwatch, and the best time of two tests was recorded. In this study, ICC was 0.988, indicating excellent test–retest reliability. In addition, the MDC value was calculated as 0.2 s, corresponding to the SEM of 0.07.

### 2.4. Exercise Protocols

#### 2.4.1. DVR Exercise

**Figure 3:**
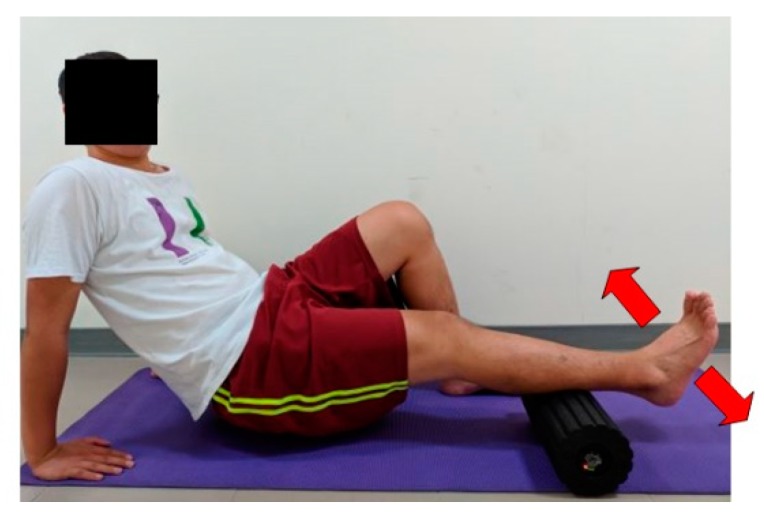
Vibration rolling combined with dynamic muscle contraction on the ankle joint.

Participants performed VR using a vibrating foam roller (dimensions: 36 × 20 × 15 cm^3^; weight: 1.8 kg) that comprised a vibration generating motor surrounded by an expanded polypropylene foam outer shell (Vyper By Hyperice, Irvine, CA, United States). First, participants positioned their right feet in the assigned position and subsequently put as much of their body weight as possible on the vibrating foam roller (frequency: 28 Hz), which is within the range of the effect of the musculoskeletal system [25]. Thereafter, they performed 30 s of moving back and forth at 40 beats per minute using a metronome while actively performing dorsiflexion and plantarflexion of their ankle (Figure 3). Subsequently, the same exercise was performed on the left foot, with 10 s of rest in between. Each exercise was performed three times.

#### 2.4.2. VR Exercise

The exercise protocols were the same as those used for the DVR exercise, except without actively performing ankle dorsiflexion and plantarflexion.

#### 2.4.3. Static Stretching

Participants performed plantarflexion in the standing position, slowly stretching the target muscle to the point of discomfort for 30 s and then performing the same exercise with the other foot. Each stretching exercise was performed three times.

### 2.5. Statistical Analyses

All data analyses were performed using SPSS, version 19 (Chicago, IL, USA). Data are presented as mean ± standard deviation (SD). Data were observed visually and statistically for normality (Shapiro–Wilk’s test, *p* > 0.05), and all variables were normally distributed. Descriptive statistics were performed for the characteristics of participants. If the sphericity assumption was violated in Mauchly’s sphericity test, the Greenhouse–Geisser adjustment was used to correct the degrees of freedom. A 2 (time: pretest vs posttest) × 3 (condition: DVR vs VR vs SS) ANOVA was performed to examine the effects of different conditions on dependent variables. If a significant condition by time interaction was found, then a full analysis of simple main effects was conducted. If a significant (*p* <   0.05) effect was observed, one-way ANOVA with Bonferroni correction for post hoc test was conducted. The effect size (Cohen’s d), which is the difference between pretest and posttest means divided by their common SD, was calculated and interpreted as small (*d* = 0.2), medium (*d* = 0.5), or large (*d* = 0.8), to present the magnitude of the effect [36]. In addition, the change in value from pretest to postintervention was calculated. The significance level (α) was considered as *p* < 0.05.

A priori sample size calculation based on anticipated differences in ankle dorsiflexion ROM as the primary outcome was estimated based on an anticipated large effect (effect size = 0.7) between pretest and postintervention. The calculation was based on an alpha level of 0.05 and a desired statistical power of 80% using G*Power [37]. The minimum sample size was 15 patients per group. In addition, assuming a dropout rate of 10% [38], we enrolled 20 participants.

To confirm that ROM measurements were greater than the measurement error, the MDC value was calculated. To calculate MDC, SEM was first calculated using the following formula: SEM = SD × √ (1-ICC), where SD is the standard deviation of scores from the first test and ICC is the test–retest ICC. Subsequently, MDC was calculated using the following formula: 1.96 × √2 × SEM [25].

## 3. Results

**Figure 4:**
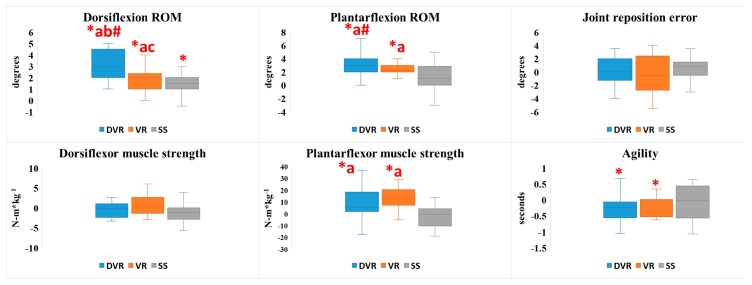
Changes from pretest to postintervention in ankle range of motion (ROM), proprioception, muscle strength, agility after the dynamic contraction with vibration rolling (DVR), vibration rolling (VR), and static stretching (SS) conditions. * Change was statistically significant at *p* < 0.05. ^a^ Significant difference (*p* < 0.05) compared with SS. ^b^ Significant difference (*p* < 0.05) compared with VR. ^c^ Significant difference (*p* < 0.05) compared with DVR. ^#^ change values reached moderate effect size.

The results of all outcomes are presented in Table 1 and Figure 4.

### 3.1. Ankle ROM Outcomes

For ankle dorsiflexion ROM, the condition × time interaction (*p* < 0.001) was significant, indicating a difference in group and time interventions. All groups showed significant improvement in post hoc test measures (*p* < 0.01) compared with pretest measures. In addition, compared with groups in change values, DVR showed more significant improvement than VR (*p* < 0.001) and SS (*p* < 0.001).

For ankle plantarflexion ROM, the condition × time interaction (*p* < 0.001) was significant, indicating a difference in groups and time interventions. All groups showed significant improvement in post hoc test measures (*p* < 0.01) compared with pretest measures. In addition, compared with groups in change values, DVR showed significant improvement compared with SS (*p* < 0.001).

### 3.2. Joint Proprioception Outcomes

For the knee joint proprioception test, the condition × time interaction (*p* = 0.909) was not significant. Compared with preintervention measures, no significant differences (DVR, *p* = 1.00; VR, *p* = 0.659; SS, *p* = 0.924) were noted. In addition, compared with groups in change values, no significant differences (*p* = 1.00) were noted.

### 3.3. Muscle Strength Outcomes

For ankle dorsiflexion peak torque, the condition × time interaction (*p* = 0.248) was not significant. No significant differences were observed in all groups (DVR: *p* = 0.724, VR: *p* = 0.131, SS: *p* = 0.081) in comparison with pretest measures; additionally, compared with groups in change values, no significant difference (*p* = 0.766) was noted.

For ankle plantar flexion peak torque, the condition × time interaction (*p* < 0.001) was significant, indicating a difference in groups and time interventions. DVR (*p* = 0.007) and VR (*p* < 0.001) significantly improved post hoc test measures compared with pretest measures. In addition, compared with groups in change values, both DVR (*p* = 0.008) and VR (*p* = 0.001) showed significant improvement compared with SS; however, no significant difference was observed between DVR and VR (*p* = 0.99).

### 3.4. Agility Outcomes

For the figure-of-8 hop test, the condition × time interaction (*p* = 0.323) was not significant. Compared with preintervention measures, a significant difference was observed in DVR (*p* = 0.016) and VR (*p* = 0.007); compared with groups in change values, no significant difference (DVR vs VR: *p* = 1.00, DVR vs SS: *p* = 0.631, VR vs SS: *p* = 0.68) was observed.

## 4. Discussion

This is the first study to investigate the immediate effects of VR combined with dynamic muscle contraction as a warm-up protocol on the ankle ROM, proprioception, muscle strength, and agility. Regarding the effects on ankle ROM, both DVR and VR significantly improved ankle dorsiflexion ROM and plantar flexion; in addition, DVR more significantly improved dorsiflexion ROM than VR (*p* < 0.001). These interventions provided comparable short-term outcomes; this is supported by previous studies [23,27]. This beneficial result may be attributable to (a) mechanical remobilization of the fascia back to its normal aligned state through SMR, and in response, soft-tissue compliance considerably increased to enable longer muscle length [39] and (b) reciprocal inhibition effects [40]. During DVR, active joint motion (i.e., alternative dorsiflexion and plantarflexion) through agonist muscle contraction may improve the effects on the antagonist tissues through modulation of muscle activity by reciprocal inhibition and other neurological pathways [14,41]. Similar findings have been reported for dynamic stretching and proprioceptive neuromuscular facilitation techniques, which possibly use similar neurologic pathways [42,43]. Regarding the effect on ankle proprioception, we found that participants had comparable outcomes for the ankle joint reposition error after DVR, VR, and SS. In summary, this study provided the first data validating that DVR and VR (28 Hz, 30 s × 3 sets on both calf muscles) do not cause ankle proprioception changes (i.e., joint reposition angle error), which is a key factor of the increased high risk of ankle sprain injury.

Next, our study revealed that both DVR and VR significantly improved ankle plantar flexor peak torque. This finding may be because VR results in the additional transmission of mechanical oscillations to the leg, which affects physiological systems such as muscle spindles, ligament proprioceptors, and joint mechanoreceptors (e.g., the Golgi tendon) [44]. Therefore, vibrations induce high activities at the muscle belly, potentially increasing the number of motor units being recruited [28,45]. Concurrently, dynamic muscle contraction may increase blood flow [29], which may boost muscle contraction activity and further modulate activity of the antagonistic muscle through reciprocal inhibition to increase flexibility [14], thereby increasing muscle strength. However, in the current study, DVR and VR did not significantly increase ankle dorsi flexor peak torque. The reason may be that tonic nociceptive stimuli resulting from SMR and VR may result in inhibitory responses from the motor cortex, resulting in both ipsilateral and contralateral inhibition [46].

Lastly, this study revealed the outcomes of the agility test after DVR and VR; this is the first study to test the efficacy of DVR and VR. We found that both DVR and VR significantly improved agility (DVR: 2.53%, VR: 1.98%), but did not significantly improve following SS. Agility is strongly related to muscle strength, speed, reaction, power, and coordination. According to the movement of the figure-of-8 hop test, the strength of the ankle plantar flexor was the most significant; therefore, we further used Pearson correlation to analyze the correlation between agility and plantar flexor strength. The results revealed a significant negative correlation (*r* = −0.626, *p* = 0.003), which might be the reason for the improvement in agility after DVR or VR.

Currently, there is no consensus on the optimal VR technique including vibration frequency and combination of rolling techniques (i.e., coupled with active joint motion during rolling). This study has some limitations that should be investigated in the future. First, this study was performed only in healthy individuals. Second, the vibrating roller was implemented at only one frequency (28 Hz); other frequencies may result in different outcomes. Third, muscle activity (e.g., electromyography) was not performed. Fourth, rolling techniques may have generated different outcomes if other muscle groups were tested. Fifth, the intensity of rolling techniques was not investigated. Different applied loadings of body weight on the target muscle during FR could have generated different outcomes [47].

## 5. Conclusions

After DVR and VR intervention, there was a significant improvement in ankle dorsiflexion and plantarflexion ROM, plantarflexor muscle strength, and agility. SS significantly improved ankle dorsiflexion and plantar flexion ROM, but did not enhance muscle strength and agility. These findings may have implications for athletes, coaches, or researchers as they suggest that performing DVR or VR increases ankle ROM, muscle strength, and agility. Therefore, practitioners may consider prescribing DVR or VR interventions as warm-up exercises when increasing ROM, muscle strength, or agility, or changing direction performance is urgent. Moreover, DVR resulted in a considerable increase in ankle dorsiflexion. Therefore, we suggest performing DVR on bilateral calf muscles immediately prior to competition because it increases ROM, which might be advantageous for improving immediate exercise performance. This information may also be useful in designing a suitable DVR prescription, and its implementation in rehabilitative and athletic practice settings. In summary, DVR appears to be a beneficial warm-up protocol to enhance exercise performance. Practitioners may consider DVR for designing more efficient and effective preperformance routines to improve exercise performances. DVR has high potential to translate into an on-field practical application. Nevertheless, further studies are required to determine the optimal warm-up regime and to compare the effects of these warm-up protocols among subjects with musculoskeletal disorders (e.g., acute or chronic ankle injuries).

## Figures and Tables

**Table 1:** Pretest and posttest descriptive results.

Parameter		Intervention	Pre	Post	Effect Size	F (*p* Value)
Ankle Range of Motion	**Dorsiflexion (degrees)**	DVR	11.1 ± 4.16	14.18 ± 3.8 *	0.78	condition × time: 22.741 (<0.001)
		VR	11.63 ± 4.23	13.48 ± 4.45 *	0.43	time factor: 103.023 (<0.001)
		SS	11.23 ± 3.73	12.58 ± 3.85 *	0.36	
	**Plantarflexion (degrees)**	DVR	51.75 ± 5.3	55.1 ± 5.45 *	0.62	condition × time: 13.125 (<0.001)
		VR	51.03 ± 5.37	53.48 ± 5.6 *	0.45	time factor: 64.668 (<0.001)
		SS	52.4 ± 5.97	53.68 ± 6.18 *	0.21	
Joint Proprioception	**Joint reposition error (degrees)**	DVR	3.5 ± 1.94	3.5 ± 1.5	0	condition × time: 0.096 (0.909)
		VR	3.75 ± 2.51	3.48 ± 1.79	0.12	time factor: 0.073 (0.79)
		SS	3.73 ± 2.7	3.78 ± 2.44	0.02	
Muscle Max Strength	**Dorsi flexors (N-m × kg^−1^)**	DVR	0.43 ± 0.11	0.44 ± 0.09	0.05	condition × time:1.447 (0.248)
		VR	0.43 ± 0.09	0.44 ± 0.1	0.14	time factor: 0.153 (0.7)
		SS	0.43 ± 0.1	0.41 ± 0.1	0.13	
	**Plantar flexors (N-m × kg^−1^)**	DVR	1.22 ± 0.39	1.32 ± 0.35 *	0.27	condition × time: 9.652(<0.001)
		VR	1.15 ± 0.33	1.28 ± 0.35 *	0.39	time factor t: 23.18 (<0.001)
		SS	1.15 ± 0.39	1.13 ± 0.35	0.05	
Agility	**Figure-of-8 Hop test (s)**	DVR	11.05 ± 2.24	10.76 ± 2.05 *	0.14	condition × time: 1.164(0.323)
		VR	11.1 ± 2.29	10.88 ± 2.36 *	0.09	time factor: 12.213 (0.002)
		SS	11.03 ± 2.06	10.96 ± 2.17	0.03	

Data reported as mean ± SD. DVR, dynamic contraction with vibration rolling; VR, vibration rolling; SS, static stretching. * Significant difference (*p* < 0.05) compared with the pretest. Effect size: *d* = M1−M2/σ_pooled_.

## References

[1] Hootman J.M., Dick R., Agel J. (2007). Epidemiology of collegiate injuries for 15 sports: Summary and recommendations for injury prevention initiatives. J. Athl. Train..

[2] Owoeye O.B.A., Palacios-Derflingher L.M., Emery C.A. (2018). Prevention of Ankle Sprain Injuries in Youth Soccer and Basketball: Effectiveness of a Neuromuscular Training Program and Examining Risk Factors. Clin. J. Sport Med. Off. J. Can. Acad. Sport Med..

[3] Thacker S.B., Gilchrist J., Stroup D.F., Kimsey C.D. (2004). The impact of stretching on sports injury risk: A systematic review of the literature. Med. Sci. Sports Exerc..

[4] McHugh M.P., Cosgrave C.H. (2010). To stretch or not to stretch: The role of stretching in injury prevention and performance. Scand. J. Med. Sci. Sports.

[5] Gil H.M., Neiva P.H., Sousa C.A., Marques C.M., Marinho A.D. (2019). Current Approaches on Warming up for Sports Performance: A Critical Review. Strength Cond. J..

[6] Behm D.G., Chaouachi A. (2011). A review of the acute effects of static and dynamic stretching on performance. Eur. J. Appl. Physiol..

[7] Cheatham S.W., Kolber M.J., Cain M. (2017). Comparison of Video-Guided, Live Instructed, and Self-Guided Foam Roll Interventions on Knee Joint Range of Motion and Pressure Pain Threshold: A Randomized Controlled Trial. Int. J. Sports Phys. Ther..

[8] MacDonald G.Z., Penney M.D., Mullaley M.E., Cuconato A.L., Drake C.D., Behm D.G., Button D.C. (2013). An acute bout of self-myofascial release increases range of motion without a subsequent decrease in muscle activation or force. J. Strength Cond. Res..

[9] Hendricks S., Hill H.N., Hollander S.D., Lombard W., Parker R. (2019). Effects of foam rolling on performance and recovery: A systematic review of the literature to guide practitioners on the use of foam rolling. J. Bodyw. Mov. Ther..

[10] Wiewelhove T., Doweling A., Schneider C., Hottenrott L., Meyer T., Kellmann M., Pfeiffer M., Ferrauti A. (2019). A Meta-Analysis of the Effects of Foam Rolling on Performance and Recovery. Front. Physiol..

[11] Jay K., Sundstrup E., Sondergaard S.D., Behm D., Brandt M., Saervoll C.A., Jakobsen M.D., Andersen L.L. (2014). Specific and cross over effects of massage for muscle soreness: Randomized controlled trial. Int. J. Sports Phys. Ther..

[12] Cheatham S.W., Kolber M.J. (2018). Does Roller Massage With a Foam Roll Change Pressure Pain Threshold of the Ipsilateral Lower Extremity Antagonist and Contralateral Muscle Groups? An Exploratory Study. J. Sport Rehabil..

[13] Su H., Chang N.J., Wu W.L., Guo L.Y., Chu I.H. (2016). Acute Effects of Foam Rolling, Static Stretching, and Dynamic Stretching During Warm-Ups on Muscular Flexibility and Strength in Young Adults. J. Sport Rehabil..

[14] Cheatham S.W., Stull K.R. (2018). Comparison of a foam rolling session with active joint motion and without joint motion: A randomized controlled trial. J. Bodyw. Mov. Ther..

[15] Houston M.N., Hodson V.E., Adams K.K., Hoch J.M. (2015). The effectiveness of whole-body-vibration training in improving hamstring flexibility in physically active adults. J. Sport Rehabil..

[16] Tseng S.Y., Hsu P.S., Lai C.L., Liao W.C., Lee M.C., Wang C.H. (2016). Effect of Two Frequencies of Whole-Body Vibration Training on Balance and Flexibility of the Elderly: A Randomized Controlled Trial. Am. J. Phys. Med. Rehabil..

[17] Bakhtiary A.H., Safavi-Farokhi Z., Aminian-Far A. (2007). Influence of vibration on delayed onset of muscle soreness following eccentric exercise. Br. J. Sports Med..

[18] Imtiyaz S., Veqar Z., Shareef M.Y. (2014). To Compare the Effect of Vibration Therapy and Massage in Prevention of Delayed Onset Muscle Soreness (DOMS). J. Clin. Diagn. Res. JCDR.

[19] Rehn B., Lidstrom J., Skoglund J., Lindstrom B. (2007). Effects on leg muscular performance from whole-body vibration exercise: A systematic review. Scand. J. Med. Sci. Sports.

[20] Cardinale M., Lim J. (2003). Electromyography activity of vastus lateralis muscle during whole-body vibrations of different frequencies. J. Strength Cond. Res..

[21] Kerschan-Schindl K., Grampp S., Henk C., Resch H., Preisinger E., Fialka-Moser V., Imhof H. (2001). Whole-body vibration exercise leads to alterations in muscle blood volume. Clin. Physiol..

[22] Lim J.H., Park C.B. (2019). The immediate effects of foam roller with vibration on hamstring flexibility and jump performance in healthy adults. J. Exerc. Rehabil..

[23] Romero-Moraleda B., Gonzalez-Garcia J., Cuellar-Rayo A., Balsalobre-Fernandez C., Munoz-Garcia D., Morencos E. (2019). Effects of Vibration and Non-Vibration Foam Rolling on Recovery after Exercise with Induced Muscle Damage. J. Sports Sci. Med..

[24] Cheatham S.W., Stull K.R., Kolber M.J. (2018). Comparison of a Vibration Roller and a Nonvibration Roller Intervention on Knee Range of Motion and Pressure Pain Threshold: A Randomized Controlled Trial. J. Sport Rehabil..

[25] Lee C.L., Chu I.H., Lyu B.J., Chang W.D., Chang N.J. (2018). Comparison of vibration rolling, nonvibration rolling, and static stretching as a warm-up exercise on flexibility, joint proprioception, muscle strength, and balance in young adults. J. Sports Sci..

[26] Garcia-Gutierrez M.T., Guillen-Rogel P., Cochrane D.J., Marin P.J. (2018). Cross transfer acute effects of foam rolling with vibration on ankle dorsiflexion range of motion. J. Musculoskelet. Neuronal Interact..

[27] Benito A.M.d., Valldecabres R., Ceca D., Richards J., Igual J.B., Pablos A. (2019). Effect of vibration vs non-vibration foam rolling techniques on flexibility, dynamic balance and perceived joint stability after fatigue. PeerJ.

[28] Cochrane D.J. (2011). The potential neural mechanisms of acute indirect vibration. J. Sports Sci. Med..

[29] Games K.E., Sefton J.M., Wilson A.E. (2015). Whole-body vibration and blood flow and muscle oxygenation: A meta-analysis. J. Athl. Train..

[30] Halperin I., Aboodarda S.J., Button D.C., Andersen L.L., Behm D.G. (2014). Roller massager improves range of motion of plantar flexor muscles without subsequent decreases in force parameters. Int. J. Sports Phys. Ther..

[31] Kelly S., Beardsley C. (2016). Specific and Cross-Over Effects of Foam Rolling on Ankle Dorsiflexion Range on Motion. Int. J. Sports Phys. Ther..

[32] Kim D.H., An D.H., Yoo W.G. (2018). Validity and reliability of ankle dorsiflexion measures in children with cerebral palsy. J. Back Musculoskelet. Rehabil..

[33] Willems T., Witvrouw E., Verstuyft J., Vaes P., De Clercq D. (2002). Proprioception and Muscle Strength in Subjects With a History of Ankle Sprains and Chronic Instability. J. Athl. Train..

[34] Halim-Kertanegara S., Raymond J., Hiller C.E., Kilbreath S.L., Refshauge K.M. (2017). The effect of ankle taping on functional performance in participants with functional ankle instability. Phys. Ther. Sport Off. J. Assoc. Chart. Physiother. Sports Med..

[35] Caffrey E., Docherty C.L., Schrader J., Klossner J. (2009). The ability of 4 single-limb hopping tests to detect functional performance deficits in individuals with functional ankle instability. J. Orthop. Sports Phys. Ther..

[36] Lakens D. (2013). Calculating and reporting effect sizes to facilitate cumulative science: A practical primer for t-tests and ANOVAs. Front Psychol..

[37] Faul F., Erdfelder E., Lang A.G., Buchner A. (2007). G*Power 3: A flexible statistical power analysis program for the social, behavioral, and biomedical sciences. Behav. Res. Methods.

[38] Krause F., Wilke J., Niederer D., Vogt L., Banzer W. (2017). Acute effects of foam rolling on passive tissue stiffness and fascial sliding: Study protocol for a randomized controlled trial. Trials.

[39] Stone J.A. (2000). Myofascial release. Athl. Ther. Today.

[40] Hamm K., Alexander C.M. (2010). Challenging presumptions: Is reciprocal inhibition truly reciprocal? A study of reciprocal inhibition between knee extensors and flexors in humans. Man. Ther..

[41] Herda T.J., Herda N.D., Costa P.B., Walter-Herda A.A., Valdez A.M., Cramer J.T. (2013). The effects of dynamic stretching on the passive properties of the muscle-tendon unit. J. Sports Sci..

[42] Mizuno T. (2017). Changes in joint range of motion and muscle-tendon unit stiffness after varying amounts of dynamic stretching. J. Sports Sci..

[43] Behm D.G., Blazevich A.J., Kay A.D., McHugh M. (2016). Acute effects of muscle stretching on physical performance, range of motion, and injury incidence in healthy active individuals: A systematic review. Appl. Physiol. Nutr. Metab. Physiol. Appl. Nutr. Et Metab..

[44] Moezy A., Olyaei G., Hadian M., Razi M., Faghihzadeh S. (2008). A comparative study of whole body vibration training and conventional training on knee proprioception and postural stability after anterior cruciate ligament reconstruction. Br. J. Sports Med..

[45] Lau W.Y., Nosaka K. (2011). Effect of vibration treatment on symptoms associated with eccentric exercise-induced muscle damage. Am. J. Phys. Med. Rehabil..

[46] Nijs J., Daenen L., Cras P., Struyf F., Roussel N., Oostendorp R.A. (2012). Nociception affects motor output: A review on sensory-motor interaction with focus on clinical implications. Clin. J. Pain.

[47] Baumgart C., Freiwald J., Kuhnemann M., Hotfiel T., Huttel M., Hoppe M.W. (2019). Foam Rolling of the Calf and Anterior Thigh: Biomechanical Loads and Acute Effects on Vertical Jump Height and Muscle Stiffness. Sports.

